# Horizontal target size perturbations during grasping movements are described by subsequent size perception and saccade amplitude

**DOI:** 10.1371/journal.pone.0264560

**Published:** 2022-03-15

**Authors:** Pablo Sanz Diez, Annalisa Bosco, Patrizia Fattori, Siegfried Wahl

**Affiliations:** 1 Carl Zeiss Vision International GmbH, Aalen, Germany; 2 Institute for Ophthalmic Research, Eberhard Karls University Tuebingen, Tuebingen, Germany; 3 Department of Biomedical and Neuromotor Sciences, University of Bologna, Bologna, Italy; 4 Alma Mater Research Institute For Human-Centered Artificial Intelligence (Alma Human AI), University of Bologna, Bologna, Italy; Universita degli Studi di Trento, ITALY

## Abstract

Perception and action are essential in our day-to-day interactions with the environment. Despite the dual-stream theory of action and perception, it is now accepted that action and perception processes interact with each other. However, little is known about the impact of unpredicted changes of target size during grasping actions on perception. We assessed whether size perception and saccade amplitude were affected before and after grasping a target that changed its horizontal size during the action execution under the presence or absence of tactile feedback. We have tested twenty-one participants in 4 blocks of 30 trials. Blocks were divided into two experimental tactile feedback paradigms: tactile and non-tactile. Trials consisted of 3 sequential phases: pre-grasping size perception, grasping, and post-grasping size perception. During pre- and post-phases, participants executed a saccade towards a horizontal bar and performed a manual size estimation of the bar size. During grasping phase, participants were asked to execute a saccade towards the bar and to make a grasping action towards the screen. While grasping, 3 horizontal size perturbation conditions were applied: non-perturbation, shortening, and lengthening. 30% of the trials presented perturbation, meaning a symmetrically shortened or lengthened by 33% of the original size. Participants’ hand and eye positions were assessed by a motion capture system and a mobile eye-tracker, respectively. After grasping, in both tactile and non-tactile feedback paradigms, size estimation was significantly reduced in lengthening (p = 0.002) and non-perturbation (p<0.001), whereas shortening did not induce significant adjustments (p = 0.86). After grasping, saccade amplitude became significantly longer in shortening (p<0.001) and significantly shorter in lengthening (p<0.001). Non-perturbation condition did not display adjustments (p = 0.95). Tactile feedback did not generate changes in the collected perceptual responses, but horizontal size perturbations did so, suggesting that all relevant target information used in the movement can be extracted from the post-action target perception.

## Introduction

In our everyday life, reaching and grasping are significant motor behaviours to interact and explore the environment. When we reach to grasp an object, the hand is pre-shaped according to object’s intrinsic properties previously perceived, like size and shape, in order to make an appropriate grasp on it [1].

In the last years, the idea of a close action-perception coupling was theorized in a common coding perspective, such as the Theory of Event Coding [2]. Within this framework, perception and action share a common representational code, which allows for efficient action planning. This common code consists of a network of features distributed across domains (such as action and perception) that can be bound together to represent common sensorimotor events. Several behavioural studies have demonstrated the existence of the bi-directional link between action and perception in terms of an “action-modulated perception” mechanism that automatically enhances relevant object features during action preparation [3–6]. For example, a relevant feature like orientation perception is enhanced during the preparation of a grasping action and not during the preparation of a pointing action for which object orientation is unimportant [7, 8]. Additionally, several other studies defined the effect of an action on perception in the detection ability of relevant features of objects [4, 5, 9–12]. The direct influence of preparation and execution of different action types on the amount of change in size perception was investigated by Bosco and colleagues [13]. This study demonstrated that the planning and execution of grasping actions significantly influences and modifies the size perception of objects compared with the planning and execution of a reaching action.

Grasping and reaching corrections to unexpected visual perturbations in target size have been widely documented [14–20], revealing correction times between 120 and 500 milliseconds in human studies [16–20]. Although not as broadly, the inference of motor action context on perception has also been investigated [8, 21, 22]. Specifically, Linkenauger and colleagues (2011) reported that object size perception can be influenced by the motor action capacity upon the object [23]. Nonetheless, later studies debated these suggestions [24, 25], arguing that these findings may be constrained by the size-contrast illusion [25, 26], which is known to be capable of affecting both size perception and grasping judgments [27]. Based on real physical targets seen in a virtual environment, changes in size perception have been reported [28]. These changes were dependent on the type of visual perturbation induced, i.e. whether it was smaller or larger with respect the actual size perceived at the end of the grasping movement. Haptic information may have contributed to size estimation variation. Proprioception is essential in natural grasping, as it provides relevant information about certain intrinsic and extrinsic object characteristics, such as orientation and size [29]. Its importance has been demonstrated in pantomime-grasping studies, which suggest that the presence of haptic feedback supports object size definition and reduces differences between pantomime and real grasping movements [30]. In fact, both proprioception and visual information are crucial to generate coordinated goal-directed motor actions.

On the basis of this evidence and considering the relevance of multisensory integration for the grasping movement experiments, in the present study, we aimed to determine whether size perception and saccade amplitude before and after grasping movements were modified by horizontal perturbations of target size during movement execution and under the presence (TF) or absence (NoTF) of tactile feedback.

Here, saccade amplitude was one of the main outcome variables. Saccade amplitude is a motor parameter that is strongly affected when target position or target size perturbations occur during saccade execution by a processing defined saccadic adaptation [31–38]. Previously, it has been demonstrated that these modifications lead to a distortion of visual localization of the target executed by hand movement or by perceptual reports [38–43]. Based on this, we hypothesized that, being size perception and saccade amplitude relevant parameters that features both motor and perceptual responses, both could be affected by target size perturbations during action execution. Also, the relevance of haptic feedback during grasping movement led us to explore the role of tactile information in the outcome variables.

## Materials and methods

### Participants

In total, twenty-one volunteers, 14 women, and 7 men, with ages ranging from 19 to 33 years (mean age of 25.05±4.08years), were involved in this experiment. The following exclusion criteria were applied: left-handed, known ocular, neurological or musculoskeletal disorders and simultaneous participation in clinical trials. Uncorrected or corrected distance visual acuity was higher or equal to 0.00 logMAR in all the subjects.

The study was approved by the Ethics Commission of the University of Bologna. Written informed consent according to the Declaration of Helsinki was obtained from each subject after a thorough explanation about the study’s risks and benefits.

### Apparatus and setup

**Fig 1:**
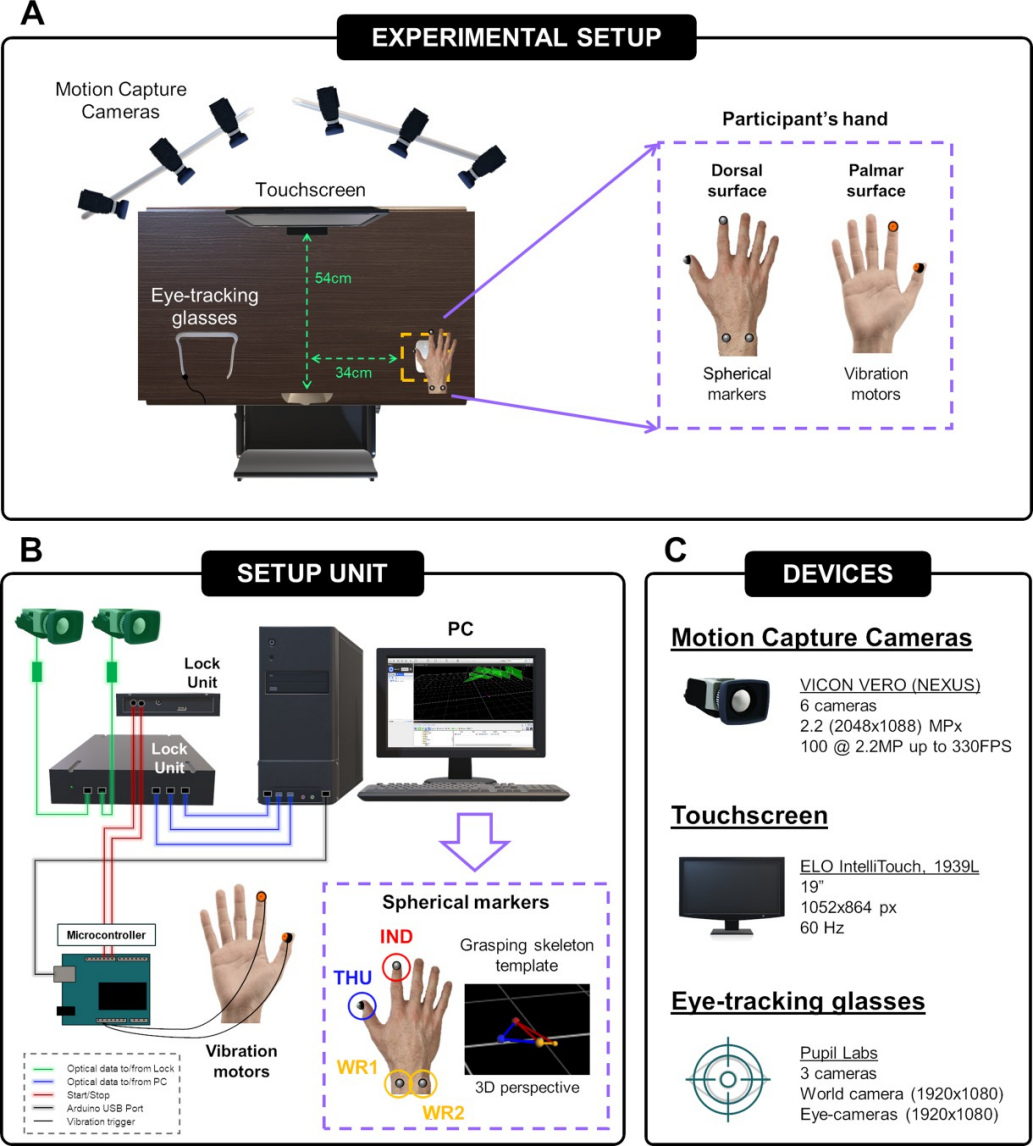
Overview of the workflow. (A) Planar view of the experimental setup. (B) Schematic diagram of the setup unit. The orthogonal optical data recorded by the motion cameras were sent to the host computer through the Lock unit. The main system used the orthogonal optical data to generate the grasping skeleton template we have created based on the position of the markers on the dorsal surface of the hand: IND (index finger), THU (thumb), WR1 (wrist, left side) and WR2 (wrist, right side). In turn, the position of the index finger and thumb markers was used in real time to activate the vibration motors through the Arduino UNO microcontroller. Arduino UNO was also responsible for triggering the start and stop VICON system capture during the experiment. (C) Characteristics of the devices used in the experiment.

Fig 1 shows a schematic representation of the experimental setup (Fig 1A) which deals with the data acquisition flow (Fig 1B) and equipment (Fig 1C) involved in the experiment. Participants sat in a dimly lit room, 54cm in front of a 19 inch touchscreen (1939L LCD, Elo Touch Solutions, Inc., Milpitas, California, United States) with 1052×864 pixels resolution and a 60Hz refresh rate (Fig 1A). A chin rest was needed to maintain the viewing distance at which the display subtended visual angles of approximately 39.8×31.8 degrees (Fig 1A).

Participant’s eye positions were recorded by a mobile eye-tracking glasses (Pupil Core, Pupil Labs GmbH., Berlin, Germany). As reported in Fig 1C, this device is composed of 3 cameras: a world camera which records the subject’s field of vision (resolution: 1920×1080 pixels; field of view: 100° fisheye; sampling rate: 60Hz) and two eye-cameras that records the observer’s eye movements (resolution: 1920×1080 pixels; sampling rate: 120Hz). Before each experimental block, a binocular calibration process was carried out automatically using five pupil calibration markers (v0.4) located in the touchscreen’s corners and centre.

Participants’s hand position and trajectory were assessed by a motion capture system, VICON VERO 2.2, with a sampling rate of 100Hz (Nexus, Vicon Motion Systems Ltd., Oxford, United Kingdom) (Fig 1C). This system is based on an actively emitting source that pulses infrared light at a very high frequency, reflected by spherical markers attached to the dorsal surface of the participant’s hand. Concretely, four spherical markers were positioned on the nail of thumb and index finger, and on the scaphoid bone of the wrist (Fig 1A). On the palmar surface of the hand, two mobile phone vibration motors (Samsung G950/G955, Samsung Co. Ltd., Seoul, South Korea) were attached to the fingerprints of the index finger and thumb to apply tactile feedback at grasp. Both the markers and the vibration motors were placed on the skin using adhesive tape (Fig 1A). Hand position at rest was delimited by a rectangular shape made with adhesive tape and located on the right side of the table, 34cm from the body midline (Fig 1A). A computer mouse located in this rectangular area was used as a trigger for the onset of the different phases of the experiment (Fig 1A). In addition, a computer loudspeaker was used to emit an auditory signal that informed the participants when to initiate the different motor and visual tasks of the experiment.

The psychophysical experiment was designed and generated in Python, using the PsychoPy software package [44]. As represented in Fig 1B, Arduino UNO microcontroller (Arduino UNO, Arduino, Milan, Italy) was responsible for setting the motor vibration frequencies and for triggering the start/stop VICON system capture. Vibration motors were set to produce square-wave with a frequency of 31Hz and 50% duty cycle. Custom Python scripts were written to unify and trigger simultaneously the beginning and end of the visual experiment with each of the different devices used.

### Behavioral task

**Fig 2:**
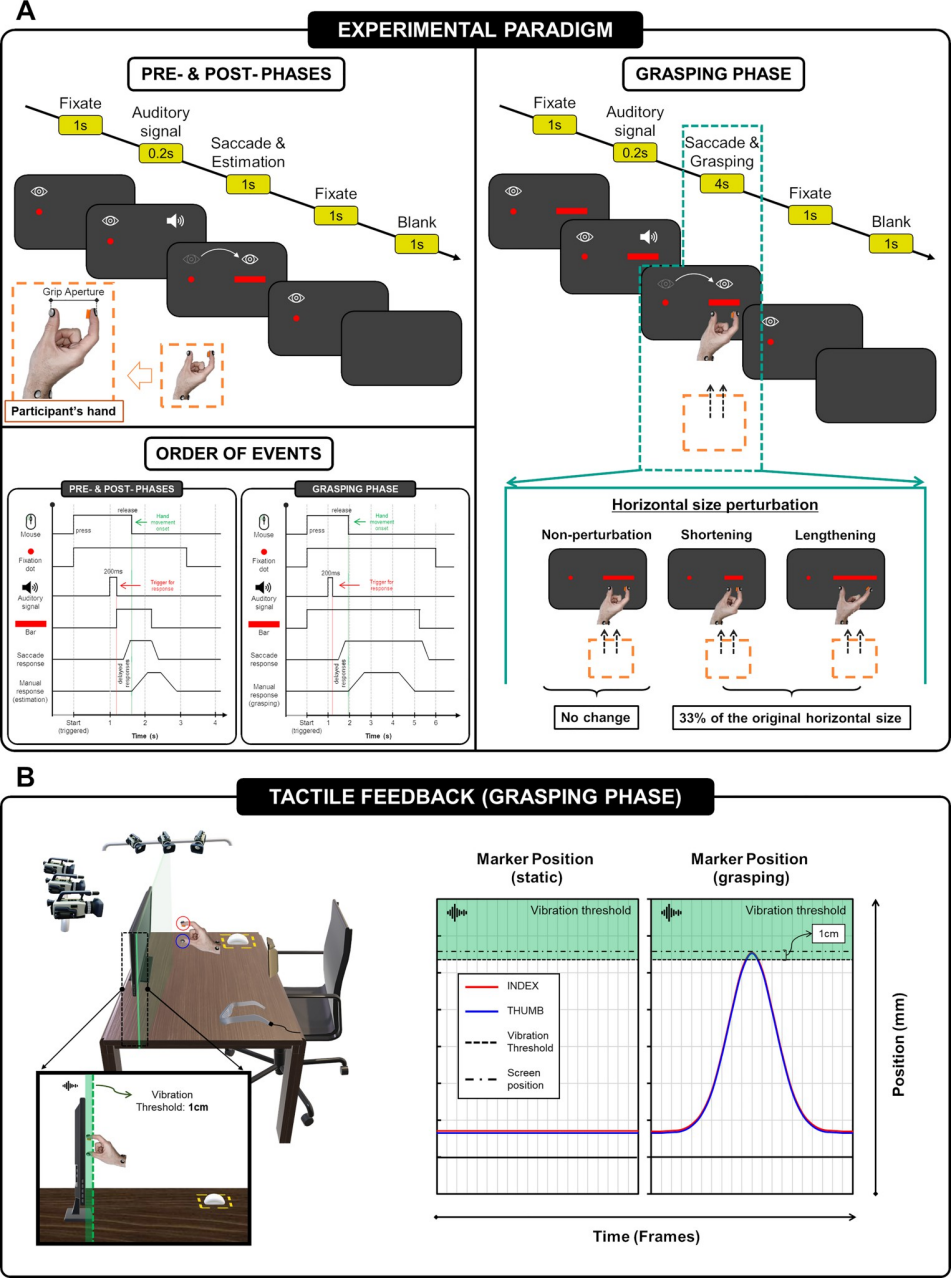
Task design. (A) Scheme of the experimental paradigm. (B) Tactile feedback implementation during the grasping phase. Tactile feedback was implemented through the coordinates of the index finger and thumb spherical markers. Once these markers were at a distance of ≤1cm from the screen, the vibration was activated. The graphs on the right show two examples illustrating the position of the markers (index finger and thumb) with respect to the screen position and the vibration threshold. Two conditions are exemplified: static position and moving position (grasping).

An illustration of the experimental paradigm can be found in Fig 2. The experimental procedure consisted of four blocks of 30 trials. These 4 blocks were divided into two categories according to the presence or absence of tactile feedback: two blocks with tactile feedback (TF) and two without it (NoTF). Block appearance order was randomized in each participant. As illustrated in Fig 2A, each trial included 3 sequential phases: pre-grasping manual estimation phase, grasping phase, and post-grasping manual estimation phase. During pre- and post- manual size estimation phases, participants were required to gaze at a red fixation dot of 0.3-degree radius located 2-degrees to the left of the screen centre for a minimum of 1s. Then, after an auditory signal of 200 milliseconds, participants had to execute a saccadic eye movement towards the horizontal bar located 12.5deg to the right of the fixation dot and to perform a manual estimation of the horizontal length of the bar. Manual size estimation consisted of indicating the horizontal size by extending the index finger and thumb. The horizontal bar target was displayed for 1s. During both manual size estimation phases, hands were out of the visual field, and therefore volunteers did not receive any visual feedback from them. In the grasping phase, as in the pre- and post- grasping manual estimation phases, participants were asked to gaze at the dot on the left side and execute a saccade movement towards the horizontal bar. However, after the auditory signal, instead of manually estimating the size, participants were instructed to make a grasping action towards the screen. For the grasping action, participants were instructed to perform a natural movement and to grasp the edges of the displayed horizontal bar by extending the index finger and thumb. Here, the horizontal bar target was displayed for a total of 5.2s. After the onset of the grasping movement, 30% of the trials presented a random horizontal size perturbation, which meant a symmetrically shortened or lengthened by 33% of the original horizontal size. Three horizontal size perturbation conditions were applied: non-perturbation, shortening, and lengthening (Fig 2A). The left mouse button was used as a trigger during the whole experimental paradigm. Specifically, the start of each one of the three sequential phases was triggered by clicking the left mouse button. The release of the left mouse button also served as a reference mark to define the start of the grasping movement.

As mentioned above, the experiment was divided into 2 categories: TF and NoTF, which were characterized by the presence or absence of tactile feedback during the grasping phase. As depicted in Fig 2B, tactile feedback was implemented by vibration, i.e., by the activation of the vibration motors placed on the fingerprints of the index finger and thumb. Vibration onset was triggered by the positioning (x, y, z) of the index finger and thumb spherical markers. The vibration was activated when these spherical markers were at a distance of ≤1cm from the screen (Fig 2B). The stimuli presented were red on a black background. Ten different sized horizontal bars were used: 3.18, 3.56, 3.94, 4.32, 4.71, 5.09, 5.47, 5.85, 6.23, 6.62 deg. All of them 0.6 deg high.

Room illumination was constant for each participant and during the whole experiment session to avoid possible disturbances in the measuring devices and the observer’s perception.

### Data processing and statistical analysis

For gaze data processing and analysis, the Pupil Player tool was used to extract gaze position data in normalized coordinates (Pupil Labs, release v1.22, Berlin, Germany). A custom MATLAB script (MathWorks, USA) was created to convert the normalized data into degrees (deg), considering viewing distance and field of view and resolution of the Pupil Labs world camera. For data filtering, only data related to confidence values greater than 0.6 were used. For data filtering, as specified by the manufacturers (Pupil Labs, Berlin, Germany), only data related to confidence values greater than 0.6 were used. Data filtering based on confidence values allowed us to detect and remove all those samples that were not relevant for the gaze analysis: blinks, noise, and corrupted values due to erroneous gaze detections. Thereafter, two categories of eye movements were identified: saccades and fixations. For saccade detection, a customized algorithm based on the eye movement velocity profile was used for the detection of saccade intervals: onset and offset. Saccade onset was determined as the point in time when the eye velocity was greater than 30 degrees/second. After onset detection, offset was defined as the moment the velocity was below 10 degrees/second. Fixations were then defined as all those gaze samples that were not classified as noise, blinks, or saccades [45]. Once the two types of eye movements were categorized, the saccade amplitudes were computed. Saccade amplitudes were calculated by subtracting fixations across 1000ms window preceding the saccade onset and within an area of interest of 0.6° (0.0° margin) and fixations across 1000ms window following the saccade offset. Time windows were equivalent to the length of time that the stimuli were displayed. Saccade amplitude was then determined as the difference between the gaze position before the saccade onset and the gaze position after the saccade offset.

For hand data processing and analysis, a custom MATLAB algorithm (MathWorks, USA) was used to compute the distance between index and thumb markers during size estimation [38]. Grip aperture values were calculated considering trial intervals in which the velocities of the index and thumb markers remained <5 mm/s [38]. Grip aperture was defined as the maximum distance within this interval. Units of measure were millimetres (mm).

Size estimation changes before and after the grasping phase were calculated as the difference of size estimation values between post-grasping and pre-grasping manual estimation phases. Likewise, the shift in saccade amplitude after the grasping phase was computed as the difference of amplitudes between post- and pre- phases.

Statistical analysis was carried out using the MATLAB R2020a statistical toolbox (MathWorks, USA) and SPSS statistical software package, version 22.0 for Windows (SPSS, Chicago, Illinois, USA). A Shapiro Wilk test was employed to assess the normality of the data. Statistical analyses were done using the appropriate tests depending on the data distribution. Multiple regression analysis was conducted to predict the length of the horizontal bar based on saccade amplitude and grip aperture values. Multinomial logistic regression was performed, with the perturbation condition as dependent outcome variable. Cox and Snell’s, Nagelkerke’s and McFadden’s goodness-of-fit tests were used to evaluate the fit of the model. Likelihood ratio tests were used to assess the overall contribution of each independent variable to the model. Differences were considered statistically significant when the associated p-values were lower than 0.05. Results are provided as mean ± pooled standard deviations.

## Results

In this study, manual size perception and saccade amplitude were evaluated before and after grasping movements towards targets that changed their horizontal size properties during the action execution. Two experimental tactile feedback paradigms, with and without tactile feedback (TF and NoTF) and three horizontal size perturbation conditions, (1) non-perturbation, (2) shortening, and (3) lengthening, were carried out to evaluate whether those perceptual responses were affected before and after grasping a variable-size target under the presence or absence of tactile information (see Figs 1 and 2 for experimental setup and paradigm).

As previously mentioned, one of the purposes of this study was to investigate whether size perception and saccade amplitude (before and after grasping movement) were affected by the presence or absence of tactile feedback. During the grasping phase, participants were asked to perform a grasping movement towards the screen and grasp the edges of the horizontal bar. In the TF condition, when participants approached the target bar, during the grasping movement they experienced a vibration in their index and thumb fingerprints exerted by mobile vibration motors. Kruskal-Wallis test followed by Fisher’s least significant difference post-hoc test revealed no significant differences between NoTF and TF neither in the size estimation adjustment (shortening: p = 0.49; non-perturbation: p = 0.29; lengthening: p = 0.98) nor in the saccade amplitude adjustment (shortening: p = 0.33; non-perturbation: p = 0.82; lengthening: p = 0.98). Accordingly, the results were analyzed conjointly, omitting the TF and NoTF grouping experimental feedback paradigms.

### Grip aperture

**Fig 3:**
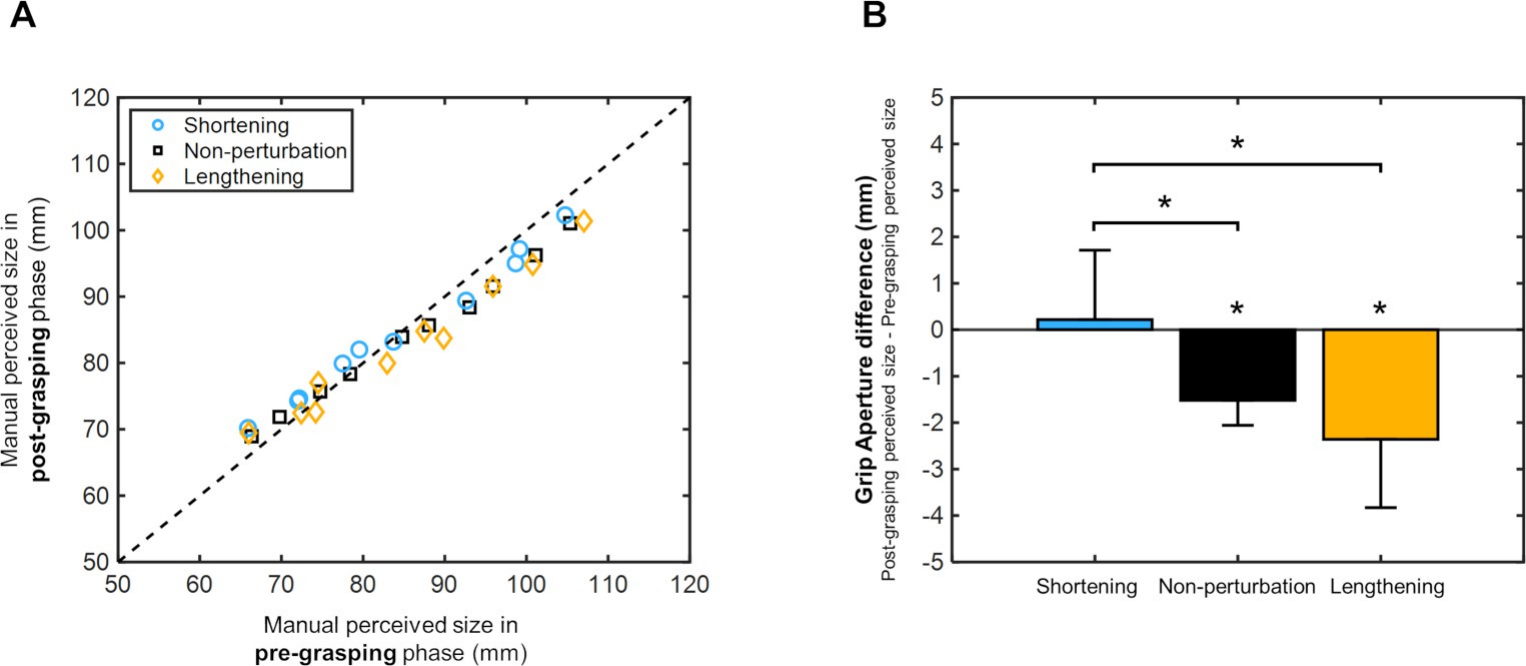
Grip aperture values during pre- and post-grasping manual estimation phases. (A) Scatter plot representing post-grasping phase perceptual responses against pre-grasping phase responses under the different horizontal size perturbation conditions: non-perturbation (black squares), shortening (blue circles) and lengthening (orange rhombuses). Reference diagonal is defined by the dashed line. (B) Difference between grip aperture values in the post- and pre- grasping phases for each size perturbation condition. Grip aperture values are shown in millimetres (mm). Error bars from the bar charts indicate pooled standard deviations. Asterisks indicate p-values less than 0.05.

Fig 3A indicates the perceptual responses under the three different horizontal size perturbation conditions. A significant positive correlation between pre- and post- manual size estimation values was observed in all horizontal perturbation conditions (Spearman correlation: r>0.98, p<0.001). For non-perturbation and lengthening conditions, most responses stayed below the diagonal line, suggesting an adjustment of perceptual size estimation after grasping movement. To assess whether this adjustment was different after the three horizontal size perturbation conditions, we calculated the amount of perceptual adjustment as the difference between the manual size estimations in the post- and pre- grasping phases. Fig 3B shows an adjustment towards negative values after the grasping movement in non-perturbation (-1.52±0.54mm), and lengthening conditions (-2.36±1.47mm), whereas an adjustment towards positive values in the shortening condition (0.22±1.49mm). Size estimation adjustment was significantly shifted from zero in the non-perturbation and lengthening (p<0.001 and p = 0.002, respectively; Wilcoxon signed rank test). Shortening did not reveal a significant adjustment (p = 0.86; Wilcoxon signed rank test), however, it showed significant differences compared to the non-perturbation and lengthening adjustments (Kruskal-Wallis test: chi-square = 6.66, p = 0.03; followed by Fisher’s least significant difference test: p = 0.017 and p = 0.02, respectively). These results indicate that, after a grasping movement, participants perceived smaller bar sizes in the lengthening and non-perturbation conditions whereas size perception was not affected in the shortening condition.

A regression analysis was conducted to evaluate the relationships between bar sizes and the manual size estimation responses in the pre- and post-grasping phases. Grip aperture responses presented a significant linear fit with the real sizes both before and after the grasping movement in all size perturbation conditions (Spearman correlation: r>0.97, p<0.001). This indicates the participants’ good capability in discerning the sizes of the bars.

**Fig 4:**
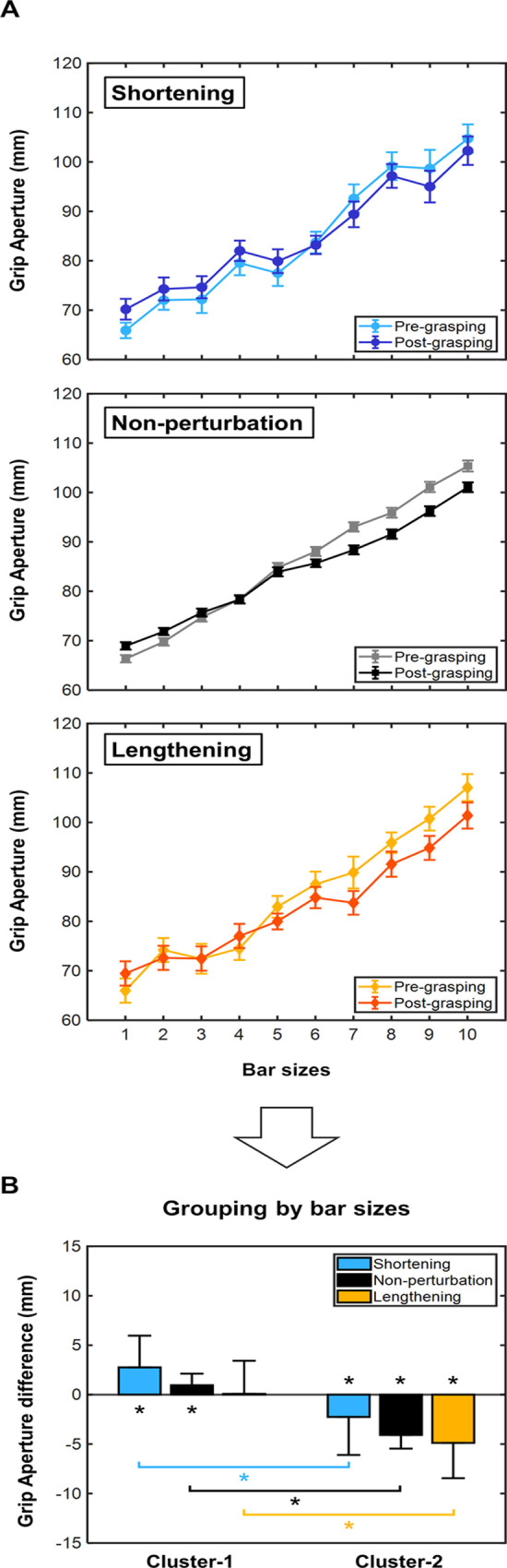
Relationship between grip aperture values and horizontal bar sizes. (A) Grip aperture values in pre- and post-grasping phases for each horizontal bar size and horizontal perturbation conditions (non-perturbation, shortening, and lengthening). (B) Grip aperture difference values clustered into 2 groups of bar sizes (Cluster-1: sizes 1 to 5; Cluster-2: sizes 6 to 10). Each bar represents the different horizontal size perturbation conditions for each size cluster: non-perturbation (black), shortening (blue) and lengthening (orange). Values from bar charts are represented as pooled mean and error bars as pooled standard deviations. All values are presented in millimetres (mm). Bar sizes from 1 to 10 (in ascending order): 3.18, 3.56, 3.94, 4.32, 4.71, 5.09, 5.47, 5.85, 6.23, 6.62 deg.

Fig 4A shows the grip aperture values in pre- and post-grasping phases for each bar size and horizontal perturbation conditions. The difference between pre- and post- phases revealed a strong inverse linear relationship between bar sizes and grip aperture difference values in all horizontal perturbation conditions (non-perturbation: r = -0.92, p<0.001; shortening: r = -0.86, p = 0.004; lengthening: r = -0.86, p = 0.004; Spearman correlation). This means that, as bar size increases, grip aperture difference values shifted towards the negative range and vice versa. In Fig 4B, two bar size clusters were generated (Cluster-1: bar sizes from 1 to 5; Cluster-2: bar sizes from 6 to 10) showing different behaviours in the post-grasping size perception. In particular, after grasping movements, for smaller horizontal bar sizes participants perceived larger sizes, whereas for larger bar sizes participants perceived smaller sizes (Fig 4B). In Cluster-1, a significant size over-estimation was observed in shortening and non-perturbation (Wilcoxon signed rank test: p = 0.002 and p<0.001, respectively), although not in lengthening condition (Wilcoxon signed rank test: p = 0.47). Cluster-2 showed a significant size under-estimation in the three horizontal perturbation conditions (Wilcoxon signed rank test: p = 0.002, p<0.001 and p<0.001, for shortening, non-perturbation and lengthening, respectively). In both, Cluster-1 and Cluster-2, no significant within-group differences were noticed (Kruskal-Wallis test: chi-square = 4.87, p = 0.09, for Cluster-1; chi-square = 5.16, p = 0.08, for Cluster-2). As shown in Table 1, statistically significant differences between Cluster-1 and Cluster-2 were identified in all horizontal perturbation conditions (Kruskal-Wallis test: chi-square = 214.73, p<0.001; Fisher’s least significant difference test: p<0.001 all three).

**Table 1:** Grip aperture difference values between pre- and post-grasping phases clustered by 2 groups of bar sizes.

	Grip aperture differences (Cluster-1)	Grip aperture differences (Cluster-2)	Pvalue (Pre- vs Post-)		Pvalue (Cluster-1 vs Cluster-2)
			Cluster-1	Cluster-2	
**Non-perturbation**	0.96±1.17mm	-4.08±1.37mm	<0.001	<0.001	<0.001
**Shortening**	2.75±3.21mm	-2.26±3.85mm	0.02	0.02	<0.001
**Lengthening**	0.08±3.35mm	-4.88±3.56mm	0.47	<0.001	<0.001

Bar size groups: Cluster-1: bar sizes from 1 to 5; Cluster-2: bar sizes from 6 to 10. Results are displayed as pooled mean difference ± pooled standard deviation. Pvalue (Pre- vs Post-) column indicates the differences between pre- and post-grasping phases in both clusters. Pvalue (Cluster-1 vs Cluster-2) column shows the differences between Cluster-1 and Cluster-2.

### Saccade amplitude

**Fig 5:**
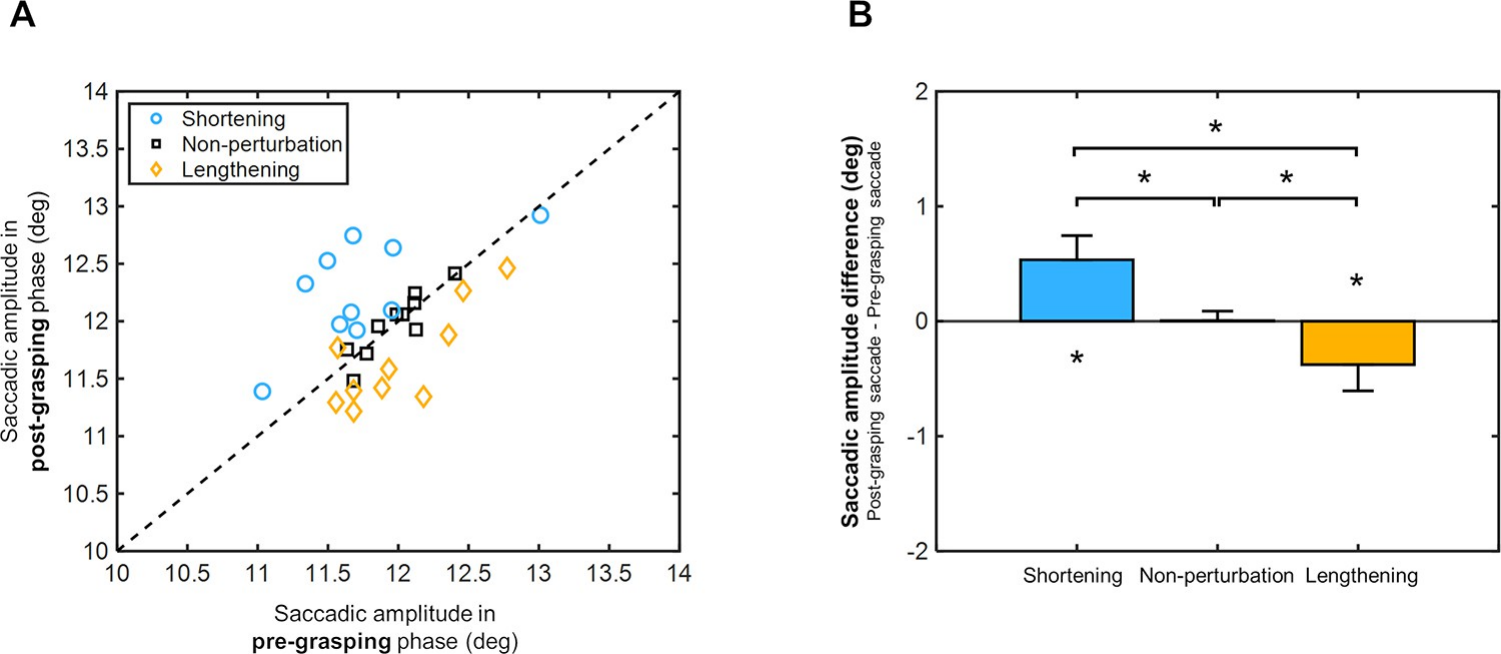
Saccade amplitude values during pre- and post-grasping manual estimation phases. (A) Scatter plots representing post-grasping phase saccades against pre-grasping phase saccades under the different horizontal size perturbation conditions: non-perturbation (black squares), shortening (blue circles) and lengthening (orange rhombuses). Reference diagonal is defined by the dashed line. (B) Difference between saccadic amplitude values inthe post- and pre- grasping phases for each size perturbation condition. Saccadic amplitude values are shown in degrees (deg). Error bars from the bar charts indicate pooled standard deviations. Asterisks indicate p-values less than 0.05.

Saccade amplitude values during pre- and post-grasping manual estimation phases are shown in Fig 5. Overall, the saccade amplitudes recorded in the non-perturbation condition show values highly concentrated on the diagonal (black squares of Fig 5A), while saccade amplitudes recorded in shortening and lengthening conditions are distributed above and below the diagonal, respectively. In addition, a significant direct relationship between pre- and post- saccade values was observed in the non-perturbation and lengthening conditions (Spearman correlation: r = 0.78, p = 0.01 and r = 0.69, p = 0.04, respectively). At the same time, not significance was found in shortening (Spearman correlation: r = 0.53, p = 0.12). Adjustment evaluation is carried out in Fig 5B, which indicates the amount of saccade adjustment as the difference between the saccade amplitude values in the post- and pre- grasping phases. As observed in Fig 5B, a significant adjustment towards positive values was found in the shortening condition (0.54±0.21deg; Wilcoxon signed rank test: p<0.001), while a significant adjustment towards negative values was detected in the lengthening condition (-0.38±0.23deg; Wilcoxon signed rank test: p<0.001). The non-perturbation condition did not display significant adjustments (0.01±0.10deg; Wilcoxon signed rank test: p = 0.95).

A within-group analysis revealed that saccade adjustments differed significantly among the three horizontal size perturbation conditions (Kruskal-Wallis test: chi-square = 51.18, p<0.001; followed by Fisher’s least significant difference test: p<0.001 among all three). The above-mentioned outcomes suggest that, after the grasping execution, participants performed larger saccade movements in the shortening condition and smaller saccade movements in the lengthening condition. As expected, linear relationships between horizontal bar sizes and saccade amplitude values in the post-grasping phases revealed a significant inverse relationship in the non-perturbation condition (Spearman correlation: r = -0.75, p = 0.02), indicating that saccade amplitude values tend to decrease linearly as bar sizes increase. Weak and non-significance linear correlations were found in the shortening (Spearman correlation: r = 0.14, p = 0.71) and lengthening conditions (Spearman correlation: r = -0.18, p = 0.63). A linear regression analysis between saccade amplitudes and manual size estimation responses after grasping movements showed a significant correlation coefficient in the non-perturbation condition (Spearman correlation: r = -0.75, p = 0.02), however such significance was not observed in shortening (Spearman correlation: r = 0.15, p = 0.68) and lengthening (Spearman correlation: r = -0.27, p = 0.45). These findings confirm that horizontal size perturbation during the grasping movement influences saccade amplitude responses after the grasping execution, i.e. during the perceptual phase of the task.

A multiple regression analysis was run to predict bar sizes from grip aperture and saccade amplitude in the post-grasping phase. The regression model significantly predicted bar sizes, F(2,27) = 335.93, p<0.001, R^2^ = 0.96. However, as shown in Table 2, only grip aperture added statistically significance to the prediction (p<0.001, t-value = -1.01), while saccade amplitude did not (p = 0.32, t-value = 25.19).

**Table 2:** Multiple regression analysis of the association between bar sizes and predictors (grip aperture and saccade amplitude).

	β	Standard Error	t-stat	Pvalue	Lower 95% CI	Upper 95% CI
**(Constant)**	-93.20	41.51	-2.25	0.03	-178.36	-8.04
**Grip aperture**	3.50	0.14	25.19	>0.001	3.22	3.79
**Saccade amplitude**	-3.18	3.14	-1.01	0.32	-9.62	3.26

A multinomial logistic regression was performed to predict the perturbation condition from grip aperture and saccade amplitude in the post-grasping phase. The final model significantly predicted the dependent variable better than the intercept-only model alone (χ^2^(4) = 12.31, p = 0.02]). Specifically, saccade amplitude was the only predictor in the model to discriminate the different perturbation conditions (χ^2^(2) = 12.10, p = 0.002), while grip aperture was not (χ^2^(2) = 1.95, p = 0.38). Considering the prediction of all three perturbation conditions, non-perturbation was correctly predicted by the model the 50% of the time. Shortening and lengthening were correctly predicted by the model the 60% and the 70% of the time, respectively.

## Discussion

This research evaluated the data on grip aperture and gaze positions in observers who were asked to make manual size estimations and saccadic eye movements before and after grasping variable-size targets under the presence or absence of tactile feedback.

We did not detect any significant modification in size perception that could be due to the presence of tactile feedback during the grasping movement. However, our data show that perceptual size reports differed significantly depending on the type of horizontal size perturbation produced during grasping execution. After grasping action in both tactile and non-tactile feedback paradigms, observers reported smaller sizes under non-perturbation conditions. These findings are consistent with those reported by Bosco et al. (2017). They observed a modification of size perception after the execution of two hand movements: grasping and reaching. Larger changes in sizes perception after the grasping movement were described. Particularly, they noted that participants perceived objects to be smaller after a grasping than after a reaching movement [13]. For both tactile and non-tactile feedback paradigms, the perceptual reports of target size after grasping movements were modified in the shortening and lengthening conditions. Size perception reduction was enhanced in the lengthening condition. In the shortening condition, observers tended to perceive equal or larger sizes after grasping execution. These data suggest that the grasping action execution modifies the perception of object size and, interestingly, these perceptual modifications depend on the changes occurring during the execution of grasping execution. Similar findings were reported by Cesanek and Domini (2018), who analyzed manual estimations after either positive or negative visual size perturbation of 7.5mm [28]. In their study, under a virtual environment, participants were instructed to grasp objects that could appear larger or smaller than the real objects detected at the end of the action execution. After negative visual perturbation (7.5-mm reduction) manual estimates were reported to be significantly larger than those following the positive perturbation (7.5-mm increase), regardless of the position of the manual judgment [28]. These findings agree with the outcomes observed in our study. As shown in Fig 3, size estimation adjustments differed significantly among the investigated perturbation conditions, showing that after lengthening perturbation, manual size adjustments were significantly smaller than those found after shortening. Concretely, Cesanek and Domini (2018) reported an average effect on the manual estimation of ~3.7mm, which implied 8% of the actual size. Instead, we found an effect of ~2.6mm, representing the 6%.

In the shortening condition, significant differences between the pre- and post-grasping phases were not observed. This absence of significant variation of size perception may suggest that perceptual size responses, after a grasping movement, are not affected equally in both directions of horizontal perturbation. Bosco et al. (2020) reported similar findings [46]. While in our study the horizontal bar size was modified during the origin of the grasping movement, in their experiment, size perturbation was based on the onset of the saccadic eye movements [46]. Different effects between shortening and lengthening conditions were reported and, as in our study, perceptual responses were not equally affected in both directions [46].

As reported in Fig 4, we found that size perception after the grasping movement was clearly influenced by bar size. In both tactile and non-tactile feedback paradigms, the comparison of pre- and post- grasping phases revealed that after the grasping act smaller bar sizes led to higher grip aperture values, while larger bar sizes resulted in smaller grip aperture values. A reason for this misperception of object size may be due to the use of two-dimensional digital objects. The accuracy of perception is clearly influenced when we evaluate the size of digitally displayed objects compared to real objects [47]. Numerous aspects differentiate real and digital objects, many of which can greatly influence our perception and even recognition [48]. Depth and shape information are critical and definitive in the comparison between the two types of objects. Even these differences have greater consequences for grasping movements than reaching movements, as real objects require more detailed preparation [49]. Our results have shown that the grasping execution modifies the subsequent size perception responses, but it does not improve perception accuracy, which may be due to the use of such digitally displayed objects. To further enhance *realism* and to compensate for the lack of tactile feedback in the two-dimensional targets, skin vibration was applied to the fingerprints at the moment of grasping execution. Under the current experimental paradigm, our results suggested that the availability of tactile feedback did not affect size perception. Similar results were reported by Park et al. (2019) when they evaluated the role of cutaneous feedback on fingerprints in the size perception of digital objects [50]. Based on touch force feedback haptic interfaces they observed that cutaneous feedback did not generate changes in size perception. However, by applying vibration to the dorsum of the hand they found that size perception was altered, showing an underestimation in size perception [50]. These results show the existing complexity for the recreation of intrinsic tactile feedback perceived in real objects, and the availability of different types of haptic feedback that can affect the size perception of two-dimensional objects in different ways.

Considering our experimental conditions and consistent with the results observed for size perception, similar effects have been found for saccade amplitude. The presence of tactile information did not generate an appreciable impact. Nevertheless, we found a modification of saccade amplitude following horizontal size perturbation during grasping movement execution. As observed in size perceptual responses, the reported impact was not strictly bidirectional, suggesting different mechanisms in the oculomotor system for different size perturbation conditions [46, 51, 52]. Several studies have investigated the plasticity of the saccadic system; observing how changes in object size during saccadic execution generate changes in amplitude, consistent with the direction in size change [33, 38, 46, 53, 54]. Although shortening and lengthening conditions were generated symmetrically, keeping the centre of gravity constant [55, 56], in the reported linear relationships we observed downward slopings, meaning that smaller objects resulted in larger saccade amplitudes, and vice versa. Furthermore, these trends were somewhat influenced under the different horizontal perturbation conditions. Comparing the pre- and post- phases, our results have shown that participants performed larger saccade movements in the shortening condition and smaller saccade movements in the lengthening condition after the execution of the grasping action. Such observed tendencies may be due to the different experimental conditions used in this study. It should be noted that unlike other investigations, we studied the saccade amplitude a posteriori, i.e., after a grasping movement which generated a change in the object size. Moreover, before the saccade onset, participants had no peripheral visual information about the object, since the object appeared just as the auditory signal ended. The time the visual stimulus was in the peripheral visual field prior to the saccadic movement was minimum. Therefore, the observed tendencies may be due to the influence exerted by visual information observed in the grasping phase. In this study, object shape was kept constant, with size being the only intrinsic aspect modified. In addition, participants were simply asked to freely gaze at the object without any specific instruction on the area where they should, so the modification of saccade amplitude could be the result of position and size information extracted from the grasping phase. In fact, we can argue that the perceptual system generates responses based on size information seen during previous motor action. This processing modifies size perception at two levels. The first level suggests that after the execution of a grasping movement, the horizontal size perception is modulated and relies on the presence of a horizontal size perturbation during the grasping action. The second level is related to saccade responses that exhibited amplitude modifications according to the types of target perturbations occurred in the previous action execution. This behavior could be ascribed to the vision of an object changing size during grasping execution and, in the subsequent perceptual phase, it is assumed the same perturbation (i.e. if the objects “grows” again, a shorter saccade is sufficient to reach it). In summary, the combination of manual perceptual reports and corresponding saccade amplitudes are descriptive parameters of previous motor actions suggesting a learning mechanism which transfers information from motor to perceptual system.
